# 17 **β** -Hydroxysteroid dehydrogenase type 1 and type 2 in human breast carcinoma: a correlation to clinicopathological parameters

**DOI:** 10.1054/bjoc.1999.0956

**Published:** 2000-01-18

**Authors:** T Suzuki, T Moriya, N Ariga, C Kaneko, M Kanazawa, H Sasano

**Affiliations:** Department of Pathology, Tohoku University School of Medicine, 2-1 Seiryo-machi, Aoba-ku, Sendai, 980–8575, Japan; Department of Pathology, Tohoku University Hospital, 1–1 Seiryo-machi, Aoba-ku, Sendai, 980–8575, Japan

**Keywords:** 17β-HSD, breast, carcinoma, human, immunohistochemistry, oestrogen

## Abstract

The expression of 17β-hydroxysteroid dehydrogenase (17β-HSD) type 1 and type 2 was examined immunohistochemically in 111 invasive ductal carcinomas, and correlated with various clinicopathological parameters. This study investigates local regulatory mechanisms of oestrogens in human breast carcinoma. 17β-HSD type 1 was immunolocalized in carcinoma cells of 68 out of 111 invasive ductal carcinoma cases (61.3%). 17β-HSD type 2 immunoreactivity was not detected in all cases examined. A significant inverse correlation was observed between the immunohistochemical expression of 17β-HSD type 1 and histological grade of the carcinoma (*P*< 0.02). There was a significant correlation between 17β-HSD type 1 and oestrogen receptor (ER) labelling index (LI) (*P*< 0.05). In addition, carcinoma cells expressing immunoreactive 17β-HSD type 1 were frequently positive for ER. 17β-HSD type 1 was also correlated with progesterone receptor (PR) LI (*P*< 0.05). There was a significant inverse correlation between 17β-HSD type 1 and Ki-67 LI (*P*< 0.0001). No significant correlations were detected between 17β-HSD type 1 and other clinicopathological parameters, including patient age, menopausal status, stage, tumour size, lymph node status and prognosis. This study suggests that 17β-HSD type 1 plays an important role in the regulation of in situ oestradiol production in hormone-dependent breast carcinomas. © 2000 Cancer Research Campaign


*British Journal of Cancer* (2000) **82**(3), 518–523
© 2000 Cancer Research Campaign
DOI: 10.1054/ bjoc.1999.0956, available online at http://www.idealibrary.com on IDEAL®

### 17β-Hydroxysteroid dehydrogenase type 1 and type 2 in human breast carcinoma: a correlation to clinicopathological parameters

**T Suzuki[1], T Moriya[2], N Ariga[2], C Kaneko[2], M Kanazawa[2] and H Sasano[1]**

[1]Department of Pathology, Tohoku University School of Medicine, 2-1 Seiryo-machi, Aoba-ku, Sendai, 980–8575, Japan; [2]Department of Pathology, Tohoku University Hospital, 1–1 Seiryo-machi, Aoba-ku, Sendai, 980–8575, Japan

**Summary** The expression of 17β-hydroxysteroid dehydrogenase (17β-HSD) type 1 and type 2 was examined immunohistochemically in 111 invasive ductal carcinomas, and correlated with various clinicopathological parameters. This study investigates local regulatory mechanisms of oestrogens in human breast carcinoma. 17β-HSD type 1 was immunolocalized in carcinoma cells of 68 out of 111 invasive ductal carcinoma cases (61.3%). 17β-HSD type 2 immunoreactivity was not detected in all cases examined. A significant inverse correlation was observed between the immunohistochemical expression of 17β-HSD type 1 and histological grade of the carcinoma ($P < 0.02$). There was a significant correlation between 17β-HSD type 1 and oestrogen receptor (ER) labelling index (LI) ($P < 0.05$). In addition, carcinoma cells expressing immunoreactive 17β-HSD type 1 were frequently positive for ER. 17β-HSD type 1 was also correlated with progesterone receptor (PR) LI ($P < 0.05$). There was a significant inverse correlation between 17β-HSD type 1 and Ki-67 LI ($P < 0.0001$). No significant correlations were detected between 17β-HSD type 1 and other clinicopathological parameters, including patient age, menopausal status, stage, tumour size, lymph node status and prognosis. This study suggests that 17β-HSD type 1 plays an important role in the regulation of in situ oestradiol production in hormone-dependent breast carcinomas. © 2000 Cancer Research Campaign

**Keywords:** 17β-HSD; breast; carcinoma; human; immunohistochemistry; oestrogen

Breast carcinoma is one of the most common malignancies in women worldwide. Human breast tissue is a target for oestrogens. Oestrogens have an important role to play in the development of hormone-dependent breast carcinomas (Thomas, 1984; Vihko and Apter, 1989), and oestrogen receptor (ER) status is well-known to affect the prognosis of patients with breast neoplasms (Rose et al, 1983). 17β-hydroxysteroid dehydrogenase (17β-HSD) catalyses the reversible interconversion of oestrogens. Recently, isozymes of 17β-HSD have been cloned, and it has been demonstrated that 17β reduction and oxidation of oestrogens are catalysed by different 17β-HSD isozymes. 17β-HSD type 1 catalyses the conversion of inactive oestrogen, oestrone (E1), to biologically active oestrogen, oestradiol (E2) (Peltoketo et al, 1988; Luu-The et al, 1989; Gast et al, 1989), while 17β-HSD type 2 catalyses the conversion of E2 to E1 (Wu et al, 1993). 17β-HSD type 1 and type 2 regulate the tissue level of E2 and modulate oestrogenic actions in oestrogen target tissues. Therefore, examining the expression of 17β-HSD type 1 and type 2 in human breast carcinoma is critical in obtaining a better understanding of the local regulation of oestrogenic actions in human breast carcinoma.

Recently, immunohistochemical studies of 17β-HSD type 1 have been reported in human breast carcinoma by Poutanen et al (1992*a*) and Sasano et al (1996). However, the number of patients examined was relatively limited in these reports, and the relationship to clinicopathological parameters and the biological significance of 17β-HSD type 1 are still unclear. In addition, little is known about 17β-HSD type 2 in human breast carcinoma. Therefore, in this study, we immunohistochemically examined the expression of 17β-HSD type 1 and type 2 in 111 human breast carcinomas, and correlated these findings with various clinicopathological parameters, in order to study the local regulatory mechanisms of oestrogens in human breast carcinoma.

### MATERIALS AND METHODS

### Patients and tissues

A total of 111 specimens of invasive ductal carcinoma of the breast were obtained from female patients who underwent mastectomy from 1984 to 1989 at the Department of Surgery, Tohoku University Hospital, Sendai, Japan. The mean age was 52 years (range 27–82). All the patients examined had not received irradiation or chemotherapy prior to surgery. The clinical data, including patient age, menopausal status, stage according to UICC TNM classification (1987), tumour size and lymph node status, were retrieved from patient charts. The histological grade in each specimen was evaluated by three of the authors (TS, TM and NA), based on the modified method of Bloom and Richardson (1957) according to Elston and Ellis (1991). The mean follow-up time was 110 months (range 13–157). Disease-free survival data were available for all patients.

All specimens were routinely processed (10% formalin-fixed and paraffin-embedded) at the Department of Pathology, Tohoku University Hospital, Sendai, Japan.

*Received 17 February 1999*
*Revised 2 August 1999*
*Accepted 2 August 1999*

*Correspondence to:* T Suzuki

**Table 1** Characteristics of primary antibodies employed in the present study

| Antibody | Source | Pretreatment | Dilution |
|---|---|---|---|
| 17β-HSD type 1 (polyclonal) | Poutanen et al., 1992a | None | 1/800 |
| 17β-HSD type 2 (monoclonal) | Moghrabi et al., 1997 | Autoclave[a] | 1/10 |
| ER (monoclonal) | Immunotech (Marseille, France) | Autoclave[a] | 1/2 |
| PR (monoclonal) | Chemicon (Temecula, CA, USA) | Autoclave[a] | 1/30 |
| Ki-67 (monoclonal) | Immunotech (Marseille, France) | Autoclave[a] | 1/50 |

[a]Heat in an autoclave for 5 min in citric acid buffer (2 mM citric acid and 9 mM trisodium citrate dehydrate, pH 6.0).

### Antibodies

The characteristics of the primary antibodies employed in this study are summarized in Table 1. 17β-HSD type 1 antibody was a rabbit polyclonal antibody against the enzyme purified from human placenta (Poutanen et al, 1992*a*), and was kindly provided by Dr Poutanen, University of Oulu, Oulu, Finland. The monoclonal antibody (mAb) of 17β-HSD type 2, mAb-C2-12, was produced by immunizing mice with a synthetic carboxyl-terminal peptide corresponding to amino acids 375–387 of 17β-HSD type 2 (Moghrabi et al, 1997), and was kindly provided by Dr Andersson, University of Texas Southwestern Medical Center, Dallas, TX, USA. Utilization of these antibodies for immunohistochemistry has been previously reported (Takeyama et al, 1998). Monoclonal antibodies for ER (ER1D5), progesterone receptor (PR; MAB429) and Ki-67 (MIB1) were purchased from Immunotech (Marseille, France), Chemicon (Temecula, CA, USA) and Immunotech (Marseille, France) respectively.

### Immunohistochemistry

Immunohistochemical analyses were performed employing the streptavidin–biotin amplification method using a Histofine Kit (Nichirei, Tokyo, Japan), and have been previously described in detail (Suzuki et al, 1994). The antigen–antibody complex was visualized with 3.3′-diaminobenzidine (DAB) solution (1 mM DAB, 50 mM Tris–HCl buffer (pH 7.6), and 0.006% hydrogen peroxide ($H_2O_2$)), and counterstained with methyl green. Tissue sections of full-term placenta were used as positive controls for 17β-HSD type 1 and type 2 (Takeyama et al, 1998). Endometrial tissue from the secretory phase was used as a positive control for 17β-HSD type 2 (Casey et al, 1994). As a negative control, normal rabbit or mouse IgG was used instead of the primary antibodies. No specific immunoreactivity was detected in these sections.

### Double immunostaining

Double immunohistochemical staining for 17β-HSD type 1 and ER was performed as previously reported (Suzuki et al, 1998). Briefly, deparaffinized sections were treated with 1% normal goat serum for 20 min, and incubated with primary antibody for 17β-HSD type 1 (dilution; 1/800) for 18 h at 4°C. The slides were then treated with alkaline phosphatase coupled anti-mouse IgG (Histofine Kit; Nichirei, Tokyo, Japan), and visualized by Vector blue (Vector Laboratories, Burlingame, CA, USA) in 0.1 M Tris–HCl buffer, pH 8.2. After pretreatment, which involved the heating of slides in an autoclave at 120°C for 5 min in citric acid buffer (2 mM citric acid and 9 mM trisodium citrate dehydrate, pH 6.0), the slides were placed in 0.3% $H_2O_2$ in methanol for 15 min, treated with 1% normal goat serum for 20 min at room temperature, and incubated with anti-ER (dilution; 1/1) for 18 h at 4°C. The slides were subsequently reacted with Envision (Dako, Copenhagen, Denmark), then visualized with DAB. Haematoxylin was used for counterstaining. As a negative control, sections were incubated with normal rabbit or mouse IgG instead of the primary antibodies. No specific immunoreactivity was detected in these sections.

### Scoring of immunoreactivity

All of the immunolabelled cells were evaluated as positive, regardless of the immuno-intensity. For statistical analyses of 17β-HSD immunoreactivity, we determined the percentage of positive cells in each carcinoma, according to the study by Sasano et al (1996), with some modification. After completely reviewing the immunostained sections of each carcinoma, three of the authors (TS, TM and NA) independently divided the carcinomas into the following three groups: ++, more than 50% positive cells; +, 0–50% positive cells; and –, no immunoreactivity. Cases with disconcordant results among the observers were re-evaluated. Scoring of ER, PR and Ki-67 in carcinoma cells was performed on high power fields (×400) using a standard light microscope. In each case, more than 500 carcinoma cells were counted independently by these same four authors, and the percentage of immunoreactivity, i.e. labelling index (LI), was determined. In the present study, inter-observer differences were less than 5%, and the mean of the three values was obtained.

### Statistical analysis

Values for patient age, tumour size and LIs of ER, PR and Ki-67 were presented as mean ± 95% confidence interval (95% CI). Association between 17β-HSD immunoreactivity and these parameters were evaluated using a Bonferroni test. We also evaluated the statistical differences between patients for 17β-HSD immunoreactivity and menopausal status, stage, lymph node status, or histological grade in a cross-table using the $\chi^2$ test. *P*-values less than 0.05 were considered as significant. Overall and disease-free survival analyses were calculated according to the Kaplan–Meier test. The statistical significance of the differences in the survival analyses was calculated using the log-rank test.

### RESULTS

### Immunohistochemistry

17β-HSD type 1 immunoreactive protein was detected in the cytoplasm of carcinoma cells (Figure 1 A,B), and the relative

© 2000 Cancer Research Campaign

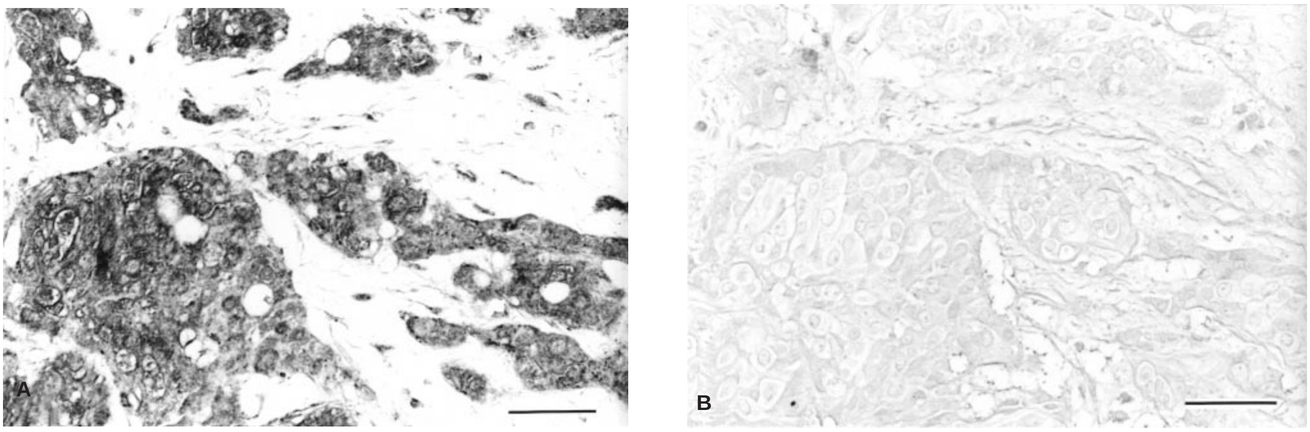

**Figure 1** Immunohistochemistry for 17β-HSD type 1 in invasive ductal carcinoma. (**A**) 17β-HSD type 1 immunoreactivity was observed in carcinoma cells. (**B**) Negative control for 17β-HSD type 1 shows no specific immunoreactivity in the same field. Original magnification ×230, bar = 50 μm respectively immunointensity was heterogeneous among the cases. In this study, the number of cases in each group according to 17β-HSD type 1 expression was as follows: ++, $n$ = 30 (27.0%); +, $n$ = 38 (34.2%); and –, $n$ = 43 (38.7%). Immunoreactivity for 17β-HSD type 2 was absent in all cases of breast carcinoma examined; however, it was detected in the vascular endothelium of the placenta and in the glandular epithelium of the endometrium during the secretory phase, as reported previously (Takeyama et al, 1998; Casey et al, 1994). Immunoreactivity for ER and PR was observed in the nuclei of carcinoma cells, and that of Ki-67 was detected in the nuclei of carcinoma cells, as well as some stromal cells.

### Correlation between 17β-HSD type 1 and clinicopathological parameters

The results of correlation between 17β-HSD type 1 and clinicopathological parameters are summarized in Table 2. Of the 111 invasive ductal carcinomas examined in the present investigation, a significant inverse correlation was detected between 17β-HSD type 1 immunoreactivity and histological grade of the carcinoma tissue ($P$ < 0.02). There was, however, no significant correlation between 17β-HSD type 1 immunoreactivity and patient age, menopausal status, stage, tumour size, or lymph node status in this study.

### Correlation between 17β-HSD type 1 and steroid receptors or Ki-67

There was a significant correlation between 17β-HSD type 1 immunoreactivity and ER LI ($P$ < 0.05; ++ (52.4 ± 10.9) vs – (33.4 ± 11.6)) (Figure 2A). Upon analysing double immunohistochemical results, 17β-HSD type 1-positive carcinoma cells were frequently positive for ER simultaneously (Figure 3 A,B). A significant correlation was also detected between 17β-HSD type 1 immunoreactivity and PR LI ($P$ < 0.05; ++ (57.7 ± 13.0) vs – (37.9

**Table 2** Correlation between 17β-HSD type 1 immunoreactivity and clinicopathological parameters in human breast carcinomas

| | 17β-HSD type 1 immunoreactivity | | | *P*-value |
|---|---|---|---|---|
| | ++ (*n* = 30) | + (*n* = 38) | – (*n* = 43) | |
| Age[a] (years) | 53.3 ± 5.1 | 54.6 ± 3.6 | 49.2 ± 3.5 | NS |
| Menopausal status | | | | |
| Premenopausal | 19 (17.1%) | 13 (11.7%) | 24 (21.6%) | |
| Post-menopausal | 11 (9.9%) | 25 (22.5%) | 19 (17.1%) | NS |
| Stage | | | | |
| I | 9 (8.2%) | 11 (10.0%) | 11 (10.0%) | |
| II | 18 (16.4%) | 19 (17.1%) | 28 (25.5%) | |
| III | 2 (1.8%) | 5 (4.5%) | 3 (2.7%) | |
| IV | 1 (0.9%) | 3 (2.7%) | 1 (0.9%) | NS |
| Tumour size* (cm) | 2.58 ± 0.62 | 2.57 ± 0.66 | 2.66 ± 0.33 | NS |
| Lymph node status | | | | |
| n: – | 15 (13.5%) | 20 (18.0%) | 23 (20.7%) | |
| n: + | 15 (13.5%) | 18 (16.2%) | 20 (18.0%) | NS |
| Histological grade | | | | |
| I | 13 (11.7%) | 9 (8.1%) | 6 (5.4%) | |
| II | 10 (9.0%) | 16 (14.4%) | 13 (11.7%) | |
| III | 7 (6.3%) | 13 (11.7%) | 24 (21.6%) | <0.02 |

Data for age and tumour size ([a]) are presented as mean ± 95% confidence interval (95% CI). All other values represent the number of cases and percentage.

 © 2000 Cancer Research Campaign

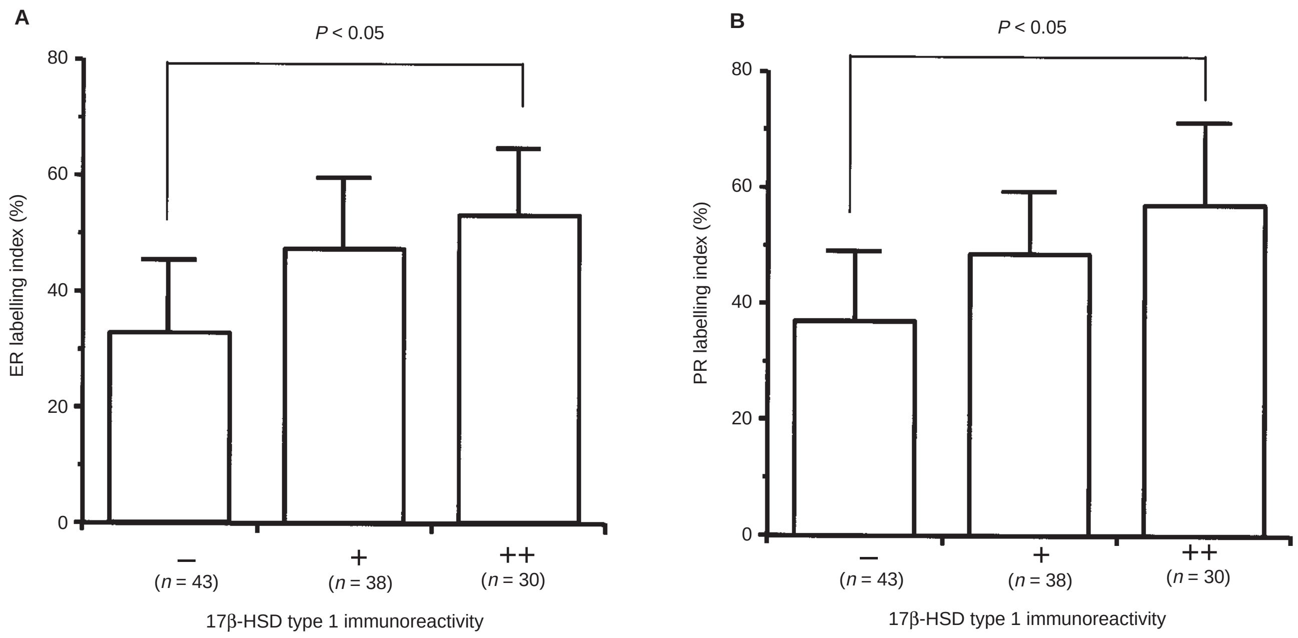

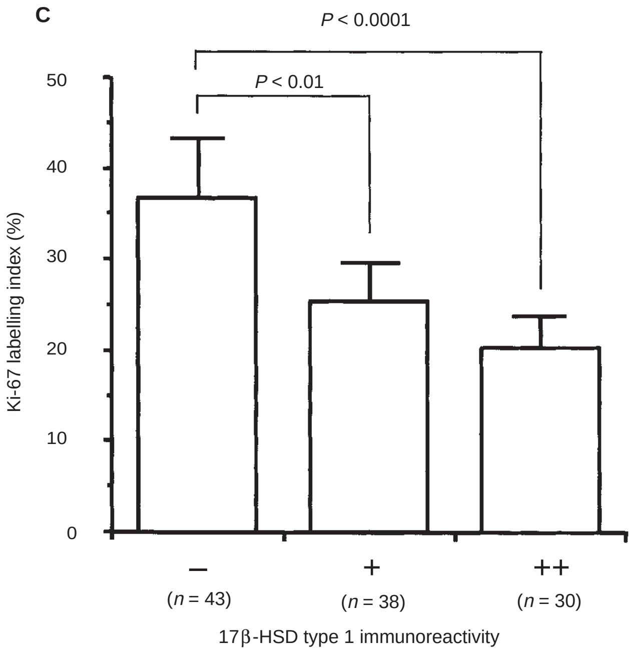

**Figure 2** Correlation between immunohistochemical expression of 17β-HSD type 1 and ER LI (**A**), PR LI (**B**), or Ki-67 LI (**C**). (**A**) There was a significant correlation between 17β-HSD type 1 immunoreactivity and ER LI ($P < 0.05$; ++ (52.4 ± 10.9) vs – (33.4 ± 11.6)). (**B**) A significant correlation was observed between 17β-HSD type 1 immunoreactivity and PR LI ($P < 0.05$; ++ (57.7 ± 13.0) vs – (37.9 ± 11.3)). (**C**) There was a significant inverse correlation between 17β-HSD type 1 immunoreactivity and Ki-67 LI ($P < 0.0001$; ++ (20.1 ± 4.6) vs – (36.8 ± 5.8), $P < 0.01$; + (25.2 ± 4.3) vs –). Data are presented as mean ± 95% confidence interval (95% CI)

± 11.3)) (Figure 2B). In this study, there was a linear relationship between ER and PR LIs ($r = 0.529$, $P < 0.0001$) when the calculation of Pearson's rank correlation coefficient was utilized.

There was also a significant inverse correlation between 17β-HSD type 1 immunoreactivity and Ki-67 LI ($P < 0.0001$; ++ (20.1 ± 4.6) vs – (36.8 ± 5.8), $P < 0.01$; + (25.2 ± 4.3) vs –) (Figure 2C).

### Correlation between 17β-HSD type 1 and prognosis

No significant correlation was detected between 17β-HSD type 1 immunoreactivity and overall or disease-free survival in the 111 invasive ductal carcinomas investigated in this study.

### DISCUSSION

It is well recognized that oestrogens contribute immensely to the development of hormone-dependent breast carcinomas (Thomas, 1984; Vihko and Apter, 1989). Previous investigations have demonstrated that tissue concentrations of E2 in breast carcinomas were more than ten times higher than those in plasma (Miller et al, 1982; Miller, 1991). However, no consistent evidence of increased serum oestrogen concentrations or other systemic oestrogen abnormalities have been observed in women with breast carcinoma (James et al, 1981). Enzymatic activities of P450 aromatase, which converts C19 steroid into E1 (Miller et al, 1982; Bulun and Simpson, 1994), and 17β-HSD (Pollow et al, 1977; Bonney et al, 1983) have been reported in breast carcinomas. In addition, P450 aromatase immunolocalization has been demonstrated in stromal cells of breast carcinoma tissues (Sasano et al, 1994). In this study, immunoreactivity for 17β-HSD type 1 was detected in carcinoma cells in 68 out of 111 invasive ductal carcinoma cases (61.3%), regardless of patient age. The frequency and localization of immunoreactive 17β-HSD type 1 are consistent with previous reports (Poutanen et al, 1992*a*; Sasano et al, 1996). These data all indicate that biologically active oestrogen E2 is synthesized from androgen precursors in situ in human breast carcinoma. In this study, immunoreactivity for 17β-HSD type 2 was absent in all cases of breast carcinoma, although the immunoreactivity was

*© 2000 Cancer Research Campaign*

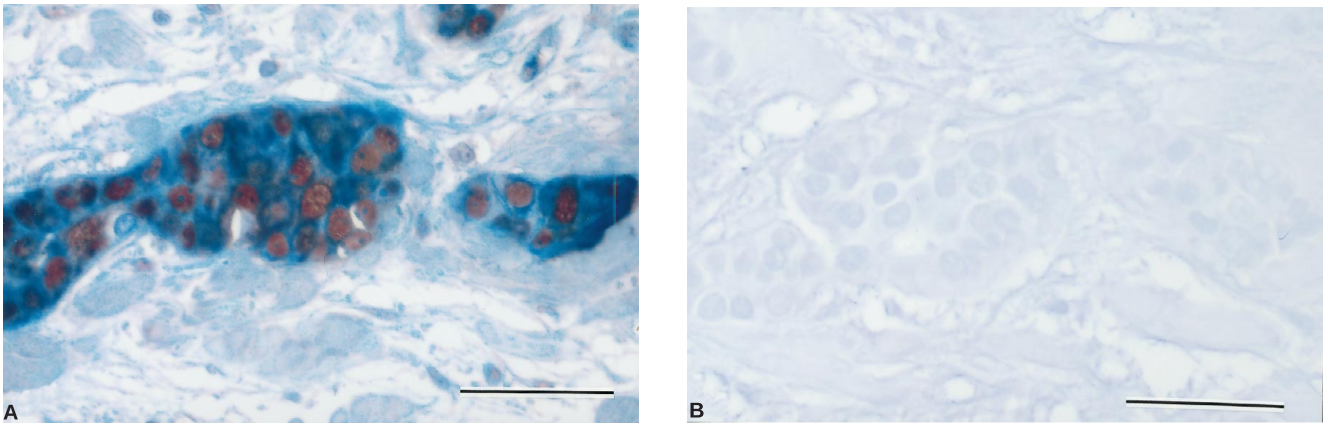

**Figure 3** Double immunostaining for 17β-HSD type 1 and ER in invasive ductal carcinoma. Blue colour represents 17β-HSD type 1, and brown colour shows ER. Haematoxylin was used for counterstaining. (**A**) The great majority of 17β-HSD type 1 immunopositive cells were also positive for ER. (**B**) Negative control shows no specific immunoreactivity in the same field. Original magnification ×400, bar = 50 μm respectively present in positive controls. Therefore, results of the present study suggest that 17β-HSD type 1 is mainly responsible for regulating the process leading to the accumulation of E2 in human breast cancer tissues. Recently, Miettinen et al (1996) reported that some breast cancer cell lines (BT-20 and MDA-MB-361) expressed 17β-HSD type 2, as well as type 1, and suggested a possible involvement of 17β-HSD type 2 in the local metabolism of oestrogens in some breast carcinomas. This discrepancy may be partly due to the heterogeneity of carcinoma cells or a difference between breast carcinoma tissues and cultured carcinoma cells.

In order to obtain a better understanding of the local regulation of E2 in human breast carcinoma, it is very important to examine the relationship between 17β-HSD type 1 and ER. Although no positive correlations have been reported previously (Fournier et al, 1985; Leszczynski et al, 1988; Sasano et al, 1996), in this study, however, a significant correlation between 17β-HSD type 1 immunoreactivity and ER LI ($P < 0.05$) was observed. In addition, co-expression of 17β-HSD type 1 and ER was frequently detected in carcinoma cells as shown in double immunohistochemical analysis. 17β-HSD type 1 immunoreactivity is suggested to reflect its enzymatic activity (Poutanen et al, 1992*a*), and ER immunoreactivity correlates closely with oestrogen-dependent biological phenomena (Marrazzo et al, 1989). Therefore, our present data suggest that E2, synthesized by 17β-HSD type 1 in carcinoma cells, acts on these cells locally in invasive ductal carcinomas. A significant correlation between 17β-HSD type 1 immunoreactivity and PR LI ($P < 0.05$) was also detected in this study, which is also consistent with a study previously reported by Poutanen et al (1992*a*). This may be partly due to the fact that the expression of PR is oestrogen-regulated (Rosen, 1996). ER-positive carcinomas are generally PR-positive (Rosen, 1996), and therefore it is not surprising to observe a strong correlation between ER and PR LIs ($r = 0.529$, $P < 0.0001$) in this study. On the other hand, it has also been demonstrated that the expression of 17β-HSD was induced by progestin in T-47D (Poutanen et al, 1990, 1992*b*) and MCF-7 (Adams et al, 1988) human breast cancer cells. Therefore, co-expression of 17β-HSD type 1 and PR may be consistent, in part, with the regulation of 17β-HSD type 1 by progesterone.

There was a significant inverse correlation between 17β-HSD type 1 immunoreactivity and Ki-67 LI ($P < 0.0001$) or histological grade ($P < 0.02$) in this study. Antibody Ki-67 has been shown to recognize cells in all phases of the cell cycle except the G0 (resting) phase (Gerdes et al, 1983), and it has been studied in a variety of normal and neoplastic human tissues (Brown and Gatter, 1990). In addition to histological grade (Elston and Ellis, 1991), Ki-67 LI is also well-known to be a useful prognostic factor in human breast carcinoma (Veronese et al, 1993). Inverse correlations between these factors and ER LI (Reiner et al, 1988; Veronese and Gambacorta, 1991) have been reported. Our present data are consistent with these previous reports, and it is suggested that breast carcinomas positive for 17β-HSD type 1 are relatively well differentiated and maintain some of the normal hormonal regulatory mechanisms that lead to a low proliferation rate. However, 17β-HSD type 1-negative carcinomas may be less differentiated and escape these controls, possibly regulating in an enhanced replicating capacity.

In summary, we demonstrated 17β-HSD type 1 immunoreactivity in invasive ductal carcinomas, but immunoreactivity of 17β-HSD type 2 was not detected in any of the cases examined. Immunoreactivity for 17β-HSD type 1 was significantly correlated with both ER and PR LIs, and inversely correlated with both Ki-67 LI and histological grade. Our present data suggest that 17β-HSD type 1 plays an important role in the regulation of in situ E2 production and actions in hormone-dependent breast carcinomas.

### ACKNOWLEDGEMENTS

This work was supported by a grant-in-aid-for Cancer Research 7–1 from the Ministry of Health and Welfare, Japan, a grant-in-aid -for scientific research area on priority area (A-11137301) from the Ministry of Education, Science and Culture, Japan, a grant-in-aid -for Scientific Research (B-11470047) from the Japan Society for the Promotion of Science, and a grant from The Naitou Foundation and Suzuken Memorial Foundation. We thank Mr. Andrew Darnel, Department of Pathology, Tohoku University School of Medicine, Sendai, Japan, for editing this manuscript.

### REFERENCES

Adams EF, Goldham NG and James VTH (1988) Steroidal regulation of oestradiol-17β dehydrogenase activity of the human breast cancer cell line MCF-7. *J Endocrinol* **118** 149–154

 © 2000 Cancer Research Campaign

Bloom HJG and Richardson WW (1957) Histological grading and prognosis in breast cancer. A study of 1409 cases of which 359 have been followed for 15 years. *Br J Cancer* **11** 359–377
Bonney RC, Reed MJ, Davison K, Beraneck PA and James VTH (1983) The relationship between 17β-hydroxysteroid dehydrogenase activity and oestrogen concentrations in human breast tumours and in normal breast tissue. *Clin Endocrinol* **19** 727–739
Brown DC and Gatter KC (1990) Monoclonal antibody Ki-67: its use in histopathology. *Histopathology* **17** 489–503
Bulun SE and Simpson ER (1994) Regulation of aromatase expression in human tissues. *Breast Cancer Res Treat* **30** 530–535
Casey ML, MacDonal PC and Andersson S (1994) 17β-hydroxysteroid dehydrogenase type 2* chromosomal assignment and progestin regulation of gene expression in human endometrium. *J Clin Invest* **94** 2135–2141
Elston CW and Ellis IO (1991) Pathological prognostic factors in breast cancer. I. The value of histological grade in breast cancer. Experience from a large study with long-term follow-up. *Histopathology* **19** 403–410
Fournier S, Brihmat F, Sterkers N, Martin PM, Kutten F and Mauvais-Jarvis P (1985) Estradiol 17β-hydroxysteroid dehydrogenase, a marker of breast hormone dependency. *Cancer Res* **45** 2895–2899
Gast MJ, Sims HF, Murdock GL, Gast PM and Strauss AW (1989) Isolation and sequencing of a cDNA clone encoding human placental 17β-estradiol dehydrogenase: identification of the putative cofactor binding site. *Am J Obstet Gynecol* **161** 1726–1731
Gerdes J, Schwab U, Lemke H and Stein H (1983) Production of a mouse monoclonal antibody reactive with a human nuclear antigen associated with cell proliferation. *Int J Cancer* **31** 13–20
James VHT, Reed MJ and Folkerd EJ (1981) Studies of estrogen metabolism in postmenopausal women with cancer. *J Steroid Biochem* **15** 235–246
Leszczynski D, Santner SJ, Feil PD and Santen RJ (1988) 17β-hydroxysteroid dehydrogenase in human breast cancer: analysis of kinetic and clinical parameters. *Steroids* **51** 299–316
Luu-The V, Labrie C, Zhao HF, Couet J, Lachance Y, Simard J, Leblanc G, Cote J, Berube D, Gagne R and Labrie F (1989) Characterization of cDNAs for human estradiol 17β-dehydrogenase and assignment of the gene to chromosome 17: evidence of two mRNA species with distinct 5′-termini in human placenta. *Mol Endocrinol* **3** 1301–1309
Marrazzo A, La Bara G, Taormina P and Bazan P (1989) Determination of oestrogen receptors with monoclonal antibodies in fine needle aspirates of breast carcinoma. *Br J Cancer* **59** 426–428
Miettinen MM, Mustonen MVJ, Poutanen MH, Isomaa VV and Vihko RK (1996) Human 17β-hydroxysteroid dehydrogenase type 1 and type 2 isozymes have opposite activities in cultured cells and characteristic cell- and tissue-specific expression. *Biochem J* **314** 839–845
Miller WR (1991) Aromatase activity in breast tissue. *J Steroid Biochem Mol Biol* **39** 783–790
Miller WR, Hawkins RA and Forrest AM (1982) Significance of aromatase activity in human breast cancer. *Cancer Res* **42** 3365–3368
Moghrabi N, Head JR and Andersson S (1997) Cell type-specific expression of 17β-hydroxysteroid dehydrogenase type 2 in human placenta and fetal liver. *J Clin Endocrinol Metab* **82** 3872–3878
Peltoketo H, Isomaa V, Maentausta O and Vihko R (1988) Complete amino acid sequence of human placental 17β-hydroxysteroid dehydrogenase deduced from cDNA. *FEBS Lett* **239** 73–77
Pollow K, Boquoi E, Baumann J, Schmidt-Gollwitzer M and Pollow B (1977) Comparison of the in vitro conversion of estradiol-17β to estrone of normal and neoplastic human breast tissue. *Mol Cell Endocrinol* **6** 333–348
Poutanen M, Isomaa V, Kainulainen K and Vihko R (1990) Progestin induction of 17β-hydroxysteroid dehydrogenase enzyme protein in the T-47D human breast-cancer cell line. *Int J Cancer* **46** 897–901
Poutanen M, Isomaa V, Lehto VP and Vihko R (1992*a*) Immunohistochemical analysis of 17β-hydroxysteroid dehydrogenase in benign and malignant human breast tissue. *Int J Cancer* **50** 386–390
Poutanen M, Moncharmont B and Vihko R (1992*b*) 17β-hydroxysteroid dehydrogenase gene expression in human breast cancer cells: regulation of expression by a progestin. *Cancer Res* **52** 290–294
Reiner A, Reiner G, Spona J, Schemper M and Holzner JH (1988) Histopathologic characterization of human breast cancer in correlation with estrogen receptor status. *Cancer* **61** 1149–1154
Rose C, Thorpe SM, Mouridsen HT, Andersen JA, Brincker H and Andersen KW (1983) Anti-oestrogen retreatment of post menopausal women with primary high risk breast cancer. *Br Cancer Res Treat* **3** 77–84
Rosen PP (1996) *Rosen's Breast Pathology*. Lippincott-Raven: Philadelphia
Sasano H, Frost AR, Saitoh R, Harada N, Poutanen M, Vihko R, Bulun SE, Silverberg SG and Nagura H (1996) Aromatase and 17β-hydroxysteroid dehydrogenase type 1 in human breast carcinoma. *J Clin Endocrinol Metab* **81** 4042–4046
Sasano H, Nagura H, Harada N, Goukon Y and Kimura M (1994) Immunolocalization of aromatase and other steroidogenic enzymes in human breast disorders. *Hum Pathol* **25** 530–535
Suzuki T, Sasano H, Kimura N, Tamura M, Fukaya T, Yajima A and Nagura H (1994) Immunohistochemical distribution of progesterone, androgen and oestrogen receptors in the human ovary during the menstrual cycle: relationship to expression of steroidogenic enzymes. *Hum Reprod* **9** 1589–1595
Suzuki T, Sasano H, Takaya R, Fukaya T, Yajima A and Nagura H (1998) Cyclic changes of vasculature and vascular phenotypes in normal human ovaries. *Hum Reprod* **13** 953–959
Takeyama J, Sasano H, Suzuki T, Iinuma K, Nagura H and Andersson S (1998) 17β-hydroxysteroid dehydrogenase type 1 and 2 in human placenta: an immunohistochemical study with correlation to placental development. *J Clin Endocrinol Metab* **83** 3710–3715
Thomas DB (1984) Do hormones cause cancer? *Cancer* **53** 595–604
Veronese SM and Gambacorta M (1991) Detection of Ki-67 proliferation rate in breast cancer. Correlation with clinical and pathologic features. *Am J Clin Pathol* **95** 30–34
Veronese SM, Gambacorta M, Gottardi O, Scanzi F, Ferrari M and Lampertico P (1993) Proliferation index as a prognostic marker in breast cancer. *Cancer* **71** 3926–3931
Vihko R and Apter D (1989) Endogenous steroids in the pathophysiology of breast cancer. *CRC Crit Rev Oncol/Hematol* **9** 1–16
Wu L, Einstein M, Geissler WM, Chan HK, Ellison KO and Andersson S (1993) Expression cloning and characterization of human 17β-hydroxysteroid dehydrogenase type 2, a microsomal enzyme possessing 20α-hydroxysteroid dehydrogenase activity. *J Biol Chem* **168** 12964–12969

© 2000 Cancer Research Campaign 
